# Simultaneous detection of chikungunya virus, dengue virus and human pathogenic *Leptospira* genomes using a multiplex TaqMan® assay

**DOI:** 10.1186/s12866-017-1019-1

**Published:** 2017-05-03

**Authors:** Claude Giry, Bénédicte Roquebert, Ghislaine Li-Pat-Yuen, Philippe Gasque, Marie-Christine Jaffar-Bandjee

**Affiliations:** 1 Centre National Arbovirus Associé, CHU de la Réunion-Site Nord, Saint-Denis, Réunion France; 2 Laboratoire de microbiologie et, CHU de la Réunion-Site Nord, Saint-Denis, Réunion France; 3 Laboratoire d’immunologie clinique et expérimentale ZOI (LICE-OI), CHU de la Réunion-Site Nord, Saint-Denis, Réunion France; 4 UMR PIMIT, Processus Infectieux en Milieu Insulaire Tropical, Université de la Réunion, INSERM 1187, CNRS 9192, IRD 249, Saint-Denis, Réunion France

**Keywords:** Chikungunya, Dengue, *Leptospira*, Multiplex real-time RT-PCR

## Abstract

**Background:**

In 2005–2006 a major epidemics of Chikungunya disease occurred in South-West Indian Ocean islands. In Reunion Island, the magnitude of Chikungunya infection related symptoms was high and with over 38% of serological prevalence in the population. This epidemics illustrated the potential threat of emerging arboviral diseases for inhabitants of Reunion Island and elsewhere since vectors are worldwide distributed. A sentinel surveillance network was set-up to detect emerging pathogens associated with fever over 38 °C and in the absence of known etiologic causes. Leptospirosis is caused by a pathogenic spirochete of the *Leptospira* genus and is an endemic and recurrent seasonal disease of great concern in Reunion Island. To accurately diagnose potentially infected patients and to advise Health authorities on the presence of emerging pathogens, a rapid diagnostic test was needed that could differentiate between these 3 pathogens.

**Methods:**

A one-step multiplex real-time PCR assay was developed that can simultaneously detect RNA of Chikungunya and Dengue viruses and leptospiral DNA with good performance for a routine diagnostic use.

**Results:**

Simplex protocols already published were used with key modifications to implement a triplex assay which was set-up with a small reaction volume to improve cost efficiency.

**Conclusions:**

This approach has enabled greater diagnostic capacity in our laboratory. We established a multiplex approach validated and valuable for cost savings, and with the concurrent detection of 3 pathogens of public health concern.

## Background

Dengue virus (DENV) and Chikungunya virus (CHIKV) belong to the genus Flavivirus and the genus Togavirus, respectively. Both are found in and transmitted by *Aedes albopictus* and *Aedes aegypti* mosquitoes. These viruses are prone to spread in the same areas and to raise similar symptoms during the acute phase of the disease [1].

CHIKV has been involved in many past and ongoing epidemics around the world. First reported in Africa as sporadic cases, epidemics of CHIKV were recorded in South East Asia, India, Western Pacific and more recently in South America [2]. Prominent features of CHIKV disease imply a suddenly appearing non-specific febrile illness with polyarthralgia, myalgia and a skin rash. Clinical signs may fade away after one week except for polyarthralgia that can persist for weeks to months [3]. CHIKV epidemics in 2005–2006 have severely impacted the Reunion population [4] with newly and unsuspected features such as *per partum* mother to infant CHIKV transmission [5] and neurological complications [6].

Dengue disease is caused by at least one of the four related dengue virus serotypes. Transmission to humans involves the bite of infected mosquitoes. The illness is variable to a large extent. Dengue fever (DF) features a non-specific acute febrile illness whereas dengue hemorrhagic fever/dengue shock (DHF/DS) syndrome is associated with hemorrhagic manifestations.

Over time, DENV have spread worldwide expanding from Southeast Asia to the Caribbean Latin America and a major epidemic was reported in Reunion Island in 1977–1978 [7]. In all these countries competent vectors are present. The status of epidemics in these countries may vary from non-endemic to hyperendemic with cases of co-circulation of multiple virus serotypes together with a risk of increased frequency of severe disease forms [8]. DF is currently a pandemic of great public health concern in the tropical countries as it is a life threatening disease for all the people in tropical and subtropical belt with a risk of exposure to DENV for 2.5 billion people, in 128 countries [9]. Since 2005, DENV circulation in Reunion Island was restricted to human cases imported from neighboring islands including Madagascar, Mayotte (type 2 and 3 DENV) or occasional travelers from Caribbean Islands (type 1 DENV) and Thailand (type 1 and 3 DENV). In 2016 an autochthonous circulation of DENV types 1 and to a lesser extent type 2 and 3 was reported Reunion Island by Health Authorities [10].

Leptospirosis is a major bacterial zoonotic disease. Human exposure to bacteria occurs accidently and involves a close contact between human skin and *Leptospira*-contaminated water. As for CHIKV and DENV, the infection is characterized by non-specific symptoms such as fever, arthralgia, myalgia and more rarely with rash [11]. Transmission to humans occurs worldwide and involves the existence of a rodent reservoir. Anthropogenic factors including human occupational or recreational activities and development of slum areas are also important in the infection process. Furthermore, in tropical areas with a hot and rainy season, leptospirosis is a recurrent disease of great health concern with significant annual incidence [12].

Hence, arboviral diseases and leptospirosis share many non-specific features such as fever, arthralgia and also thrombocytopenia in the acute phase. The broad spectrum of clinical presentations varying from asymptomatic to highly severe forms urges for the need to have differential diagnosis tests with high sensitivity and specificity. Serological diagnosis is based upon IgM detection and conversion of IgM to IgG specific antibodies. In primary infections, IgM is essentially a useful marker for diagnosis in the acute phase. In Flavivirus infections, IgG are known to cross react with other Flaviviruses impairing specificity [13]. Several simplex qPCR-based assays have been developed for either CHIKV, DENV or pathogenic *Leptospira* genome detection using a broad variety of gene targets and fluorescence formats [14, 15].

The aim of this study was to assess experimental conditions for a multiplex qPCR assay allowing simultaneous detection of the 3 targets using three sets of primers and three fluorophore-labeled probes. The design of primers and probes was optimized with low degeneracy primers and the requirement of Locked-Nucleic Acid (LNA™) -modified probes. This approach was validated according to French COFRAC SH GTA 01 – Rev. 00–05/2011 guidelines and used for two years with success in a clinical setting at the University Hospital of Reunion Island.

## Methods

### Reference samples

DENV strains 1 to 4 were obtained from National Reference Laboratory for Arbovirus of Marseilles (France) and CHIKV strain clone #4 was isolated from a clinical sample in our laboratory during CHIKV outbreak that occurred in Reunion Island in 2005. Leptospiral strains were obtained from National Reference Laboratory for Spirochetes of Paris (France) and consisted of a panel of leptospiral specimen related to pathogenic serovars (Australis, Canicola, Grypothyphosa, Hardjo, Icterohaemorrhagiae, Mini, Panama, Pyrogenes, Sejroe, Wolfi) or to a non-pathogenic serovar (Patoc*).*

### Nucleic Acid isolation

Total nucleic acids were extracted from 200 μL aliquots of human plasma samples or supernatant cell cultures using Nuclisens reagents and EasyMAG nucleic acid isolation platform according to manufacturer’s recommendations. Final elution was done in 50 μLof nuclease free water.

### Design of primers and TaqMan® probes

Simplex assays already validated and routinely used in our laboratory were chosen for multiplexing. In brief, we used CHIKV unmodified protocol from Pastorino et al. [16] targeting E1 viral gene. Pathogenic *Leptospira* was detected using a portion of rRNA 23S as target according to Woo et al. [17]. Woo originally designed his assay using two hybridization probes [17]. In order to convert Woo’s assay to an hydrolysis probe format we selected forward and reverse primers to shorten the size of the amplicon and we designed a TaqMan® probe with LNA nucleotides to reduce the length of the probe. For DENV detection, an assay targeting the 3′ non coding region (3′NC) of DENV was chosen according to Leparc-Goffard et al. [18] with two modifications at the primer and probe levels. Primers with degenerate nucleotides as less as possible were selected. These primers delineated an amplicon with a consensus sequence shared by DENV strains of the 4 types. We designed within the amplified region a 14 nucleotide probe with LNA™ modifications to fit thermal requirement of a TaqMan® probe.

DENV genomic sequences were available from Virus Pathogen Database and Analysis Resource [19]. Sequence alignment was performed with MUSCLE tool provided by VIPR. Sequence annotation was done using BioEdit Sequence Alignment Editor v7.1.3 [20]. Melting temperature and complementarity of primers were checked with Oligocalc calculator [21]. LNA™ probes were designed with LNA™ oligo tools from Exiqon and synthesized by Eurogentec®.

Due to perfect match with at least 13 nucleotides between sequences template and DRa primer, DRa likely accounts for DENV1, DENV2, DENV3 RT-PCR amplification in combination with forward primer. DRb is more specific to DENV4 template.

### One-step real time RT-PCR

Simplex assays for CHIKV and DENV were performed in a 20 μL reaction volume made of 5 μL of template and 15 μL of a mastermix containing 2× SuperScript. III Platinum® One-Step Quantitative RT-PCR System (Invitrogen). Primers and probe were purchased from Eurogentec® and used at a final concentration of 500 μM and 250 μM, respectively. Thermal cycling involved following steps: 45 °C, 15 min for reverse transcription, 95 °C, 2 min for activation and 45 cycles comprising 95 °C, 15 s, 57 °C, 5 s and 60 °C, 30 s with fluorescence acquisition on FAM channel.

Simplex assay for *Leptospira* was done in a 25 μL volume using 5 μL of template and 20 μL of a mastermix containing 2× TaqMan® Universal PCR Master Mix (Applied Biosystems), primers and probe at final concentration of 500 μM and 250 μM, respectively. Thermal cycling was as follows: 95°, 5 min and 45 cycles comprising 95°, 10 s and 60°, 50 s with fluorescence reading using FAM channel.

Multiplex assay was performed in a total volume of 10 μL including 2.5 μL of template, 2.5 μL of a 4× mix including primers and probe for CHIKV, DENV and *Leptospira*, 2.5 μL of molecular grade water and 2.5 μL of 4× ABI TaqMan® Fast Virus 1-Step Master Mix (Applied Biosystems). Final concentration of primers and probes were the same as in the simplex assays except for *Leptospira* with primers and probes reduced to half concentration. Cycling conditions were: 45 °C, 5 min, 98 °C, 20 s and 45 cycles comprising 2 steps, 98 °C, 3 s and 58 °C, 45 s with fluorescence reading using FAM, HEX and ATTO647 channels for detection of CHIV, DENV and *Leptospira*, respectively. RT-PCR cycling was set on a Roche LC480 thermal cycler (Roche Applied Science). For multiplex assay, a color compensation file was generated accordingly to Roche guidelines and used for data analysis.

### Evaluation of multiplex real time RT-PCR assay

Multiplex assay was compared to simplex assays using several approaches: determination of PCR efficiency using 10-fold dilutions of a positive control, use of a validation panel of clinical samples for Bland-Altman analysis of difference versus average of the 2 measurements of each sample, intra-assay repeatability using 15 replicates for low level and high level samples, inter-assay repeatability with 10 replicates of the same sample measured over time and accuracy measured against reference samples. Coefficient of variation (CV) was used to assess concordance between multiplex and simplex methods.

### Statistical analysis

Tests comparison was assayed for statistical significance using two-tailed Student test with an α-risk set at 0.05 using GraphPad Prism v5.0 for Windows (GraphPad Software).

## Results

### DENV assay design

As shown in Table 1, DENV reverse primer used by Leparc-Goffard was changed to 2 reverse primers in order to decrease degeneracy [18]. Alignment of representative sequences for all types of DENV allowed location of primers on DENV sequences. From in silico analyses we speculated that forward primer DF with reverse primer DRa could improve the assay with amplification of DENV1, 2 and 3. DENV4 would be better amplified with DF and DRb primers.

**Table 1:** List of primers

Name	Fluorochrome	Sequence (5′➔ 3′)	Quencher	Nt	Tm (°C)	Target
CHIK-F		AAGCTYCGCGTCCTTTACCAAG		22	62.1–64.2	E1
CHIK-R		CCAAATTGTCCYGGTCTTCTT		21	57.5–59.5	
CHIK-P	FAM	CCAATGTCYTCMGCCTGGACACCTTT	BHQ1	26	67.9–71.1	
DF		AGGACTAGAGGTTAGAGGAGA		21	59.5	
DRa		CGTTCTGTGCCTGGAATGAT		20	58.4	
DRb		CGCTCTGTGCCTGGATTGAT		20	60.5	
DP	HEX	CCA + GA + GAT + CCT + GCT	BHQ1	14	43.7 + LNA = 67	3′NC
Lep23S-F		AGAATTGGGATGAGGTGTGGATAG		24	63.6	
Lep23S-R		CTACCCCCGCAACTAAACAACTG		23	64.6	
Lep23S-P	ATTO647N	CCG + AAA + TAG + GTT + TA + GG + CCT	BHQ3	19	55 + LNA = 72	23S rRNA

Variable melting temperature was indicated for degenerate primers. Y accounts for C/T, M for A/C. Probes are quenched using adequate Black Hole Quenchers (BHQ). Locked Nucleic Acid nucleotides (LNA) are prefixed with a “+” sign and the resulted increase in Tm was indicated following use of Exiqon™ tool for calculation

**Fig. 1:**
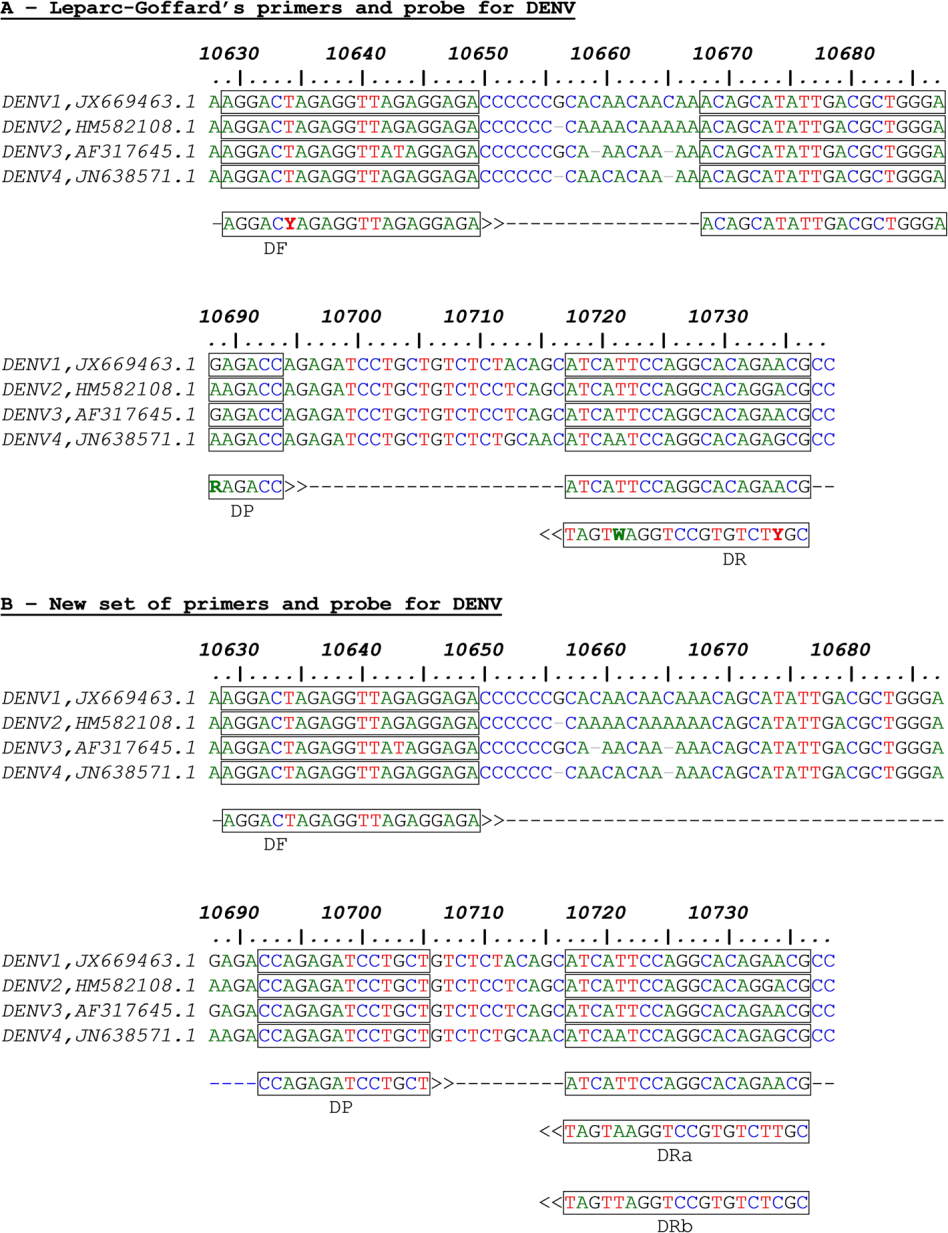
DENV sequence alignments. Genome positions refer to DENV-1 JX669463.1 sequence. *Arrows* indicate the 5′➔3′ orientation. Location of forward primer (DF), reverse primers (DRa, DRb) and probe (DP) used for generic DENV RT-PCR is indicated in *boxes* **a**) Leparc-Goffard's primers and probe for DENV. **b**) New set of primers and probe for DENV

Interestingly, the resulting amplicon for all types of DENV exhibited a consensus sequence of 14 nucleotides as shown in Fig. 1. Given the absence of mismatches between DENV sequences of all types at this location we were inclined to design a new hydrolysis probe in replacement of the original probe used by Leparc-Goffard [18]. Given that the consensus sequence amplified in our assay was very small, we made use of LNA™ nucleotides to increase the melting temperature of the reaction. The sequence of the new DENV probe was used in a blast analysis against several selected DENV genome sequences of all types. Due to the location of the primers and probe in the 3′NC region of DENV, this analysis was restricted only to complete genome sequences comprising at least 10,720 nucleotides (Table 2).

**Table 2:** Blast analysis of the new DENV probe

	Number of sequences > 10,720 nucleotides	Number of sequences perfectly matched for our dengue probe	% of sequences perfectly matched for our dengue probe
DENV1	269	266	98.9
DENV2	333	330	99.1
DENV3	159	159	100
DENV4	46	46	100

Our newly designed probe was homologous to 98.8 to 100% of the blasted sequences for Dengue type 1 to 4.

### Tests for primers cross-reactivity

Cross-reactivity of primers used in our triplex assay was checked with a panel of pathogens comprising Alphavirus (Sindbis Virus, Ross River Virus, Semliki Forrest Virus, Onyong Onyong Virus), Flavivirus (West Nile Virus, Yellow Fever Virus, Zika Virus, Hepatitis C Virus), fungi (Candida albicans) and bacteria including *Branhamella catarrhalis, Citrobacter diversus, Haemophilus parainfluenzae, Klebsiella oxytoca, Mycobacterium tuberculosis, Neisseria sp., Pseudomonas aeruginosa, Salmonella typhi, Staphylococcus aureus, Streptococcus pneumonia.* We found no evidence of cross-reaction targeting these pathogens (data not shown).

### Evaluation of PCR efficiency

PCR efficiency evaluates the duplication rate of amplicons at each cycle. It is assumed that high performance PCR systems should exhibit PCR efficiency varying from 1.85 to 2.15. We measured PCR efficiency for each target using serial dilution of a positive control and slope determination in multiplex versus simplex assays. PCR efficiency was validated and showed concordant values between assays as shown in Table 3.

**Table 3:** PCR Efficiency

	Simplex Assay			Multiplex Assay		
	CHIKV	DENV	*Leptospira*	CHIKV	DENV	*Leptospira*
Slope	−3.13	−3.19	−3.25	−3.41	−3.15	−3.37
Efficiency	2.09	2.06	2.03	1.96	2.08	1.98

Efficiency = 10(−1/slope)-1

### Test for the specificity of the assay

Specificity of our multiplex assay was checked with a panel of leptospiral specimen including pathogenic serovars (Australis, Canicola, Grypothyphosa, Hardjo, Icterohaemorrhagiae, Mini, Panama, Pyrogenes, Sejroe, Wolfi) or a non-pathogenic serovar (Patoc*).* RNA samples of the four DENV reference types were used for validation of our newly designed DENV probe. No discordance was observed between multiplex and simplex assays (data not shown).

### Bland-Altman analysis

Human plasma samples positive for the genomes of CHIKV, DENV or pathogenic *Leptospira* were used to analyze differences between multiplex and simplex assays. We first averaged the difference in Ct values obtained with simplex assay versus multiplex assay with an averaged ΔCt = Σ_i=1 to n_ (Ct_simplex-i_-Ct_multiplex-i_)/n. We found ΔCt_CHIKV_ = −1.20 ± 0.76 with *n* = 12 (two-tailed *p*- value <0.001 at the α risk of 0.05).

CHIKV assay performed slightly better in a simplex format. For DENV, ΔCt_DENV_ = +1.33 ± 0.56 with *n* = 14 (two-tailed *p* value <0.001 at the α risk of 0.05). DENV was preferentially amplified with multiplex assay resulting in reduction of 1 to 2 Cts. For *Leptospira*, ΔCt_LEPTO_ = 0.34 ± 2.17 with *n* = 21 (two-tailed *p* value >0.05, not statistically different). Both assays performed equally well for the detection of leptospiral genome.

**Fig. 2:**
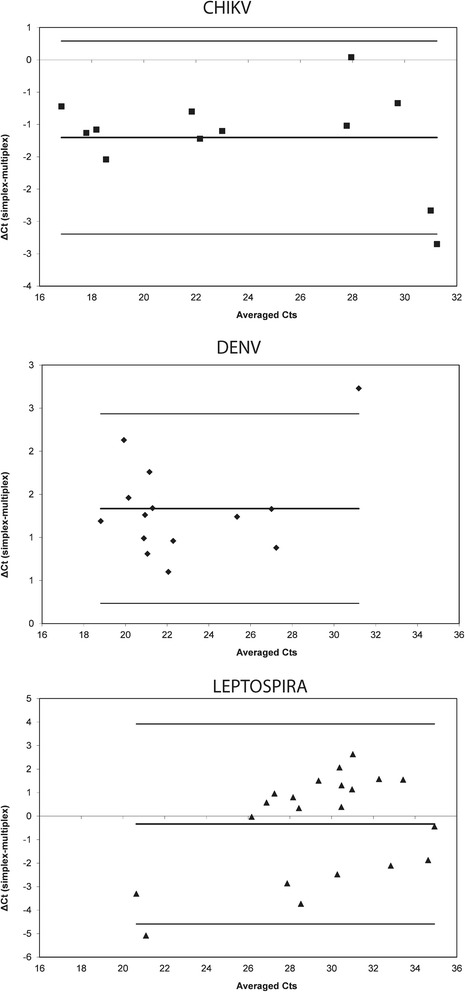
Bland-Altman plot of differences between assays. The Bland–Altman method calculates the mean difference (*bold line*) between simplex assay and multiplex assay, and 95% limits of agreement as the mean difference (*thick lines*).The Bland-Altman analysis was performed for CHIKV (*plain square*), DENV (plain rhombus) and *Leptospira* (plain triangle)

Next, we plotted on a Bland-Altman graph the differences of Ct against averaged Ct values obtained for each target and in order to check for relative agreement of simplex versus multiplex assays (Fig. 2). A confidence interval for the mean difference was chosen as follows (−1.96xSd; +1.96xSd) corresponding to a 95% probability to obtain the mean differences into these limits. This condition was validated for the 3 pathogen targets. For all targets, points were distributed according to the 95% limits of agreement.

### Clinical use of multiplex assay

This multiplex assay was routinely carried out in our laboratory to address the etiological diagnosis of dengue-like syndromes in presence of unexplained fever over 38 °C. From the period of August 2014 to July 2016, screenings were performed for suspected cases for CHIKV, DENV or leptospiral infection and leading to following results in 3028 tested cases: CHIKV 0/302/ (0%), DENV 69/3028 (2.3%), Leptospira 71/3028 (2.3%).

### Intra and inter assay variation

Intra assay variation was evaluated using coefficient of variation (CV) calculated from 15 replicates in the same assay of a low level positive control and a medium level positive control of each target. As shown in Table 4, each CV was under 5% which fits with our threshold requirement for assay validation. Inter assay variation was checked by replicating 10 times a single measurement over a period of one month. The different CV for CHIKV, DENV and *Leptospira* were 0.79, 1.29 and 1.30%, respectively.

**Table 4:** Intra assay variation

	N	Mean	SD	CV (%)
CHIKV				
Level 1	15	22.56	0.42	1.9
Level 2	15	29.24	0.44	1.5
DENV				
Level 1	15	28.48	0.36	1.3
Level 2	15	35.71	0.75	2.1
*LEPTOSPIRA*				
Level 1	15	21.74	0.97	4.4
Level 2	15	28.13	0.54	1.9

Level 1 accounts for medium amount of target, level 2 for low amounts of target

A threshold of 5% maximum for CV was required for assay validation

### Multiplex assay accuracy for CHIKV and DENV

Accuracy of our multiplex assay was checked using external quality assessment standards (EQA) from Integrated Quality Laboratory Services (IQLS) in charge of implementing the SEGA network (epidemiological surveillance and response in the Indian Ocean). Bias was calculated against data produced by the reference laboratory or against the averaged data of all laboratories enrolled in the SEGA network. A negative bias against other laboratories accounts for better sensitivity of our test compared to others. Our CHIKV results have shown similar performance with the reference laboratory and an increased sensitivity of 7.91% compared to all laboratories.

For DENV detection, our assay was characterized by an increased sensitivity of 8.05% compared to the reference laboratory and produced similar results when compared to other laboratories (Table 5).

**Table 5:** Multiplex assay accuracy for CHIKV and DENV

EQA ID	Our laboratory multiplex assay Ct	Reference laboratory assay Ct	All enrolled laboratory in SEGA network Ct	Bias against reference laboratory	Bias against all enrolled laboratories in SEGA network
CHIKV					
EQA-SEGA-05-01	23.98	23.19	26.53	+3.41%	-9.61%
EQA-SEGA-05-05	25.04	26.50	26.70	- 5.51%	-6.22%
EQA-SEGA-05-08	22.94	22.06	24.93	+3.99%	−7.98%
Mean	23.99	23.92	26.05	+0.29%	-7.91%
DENV					
EQA-SEGA-05-03	27.51	30.13	28.28	−8.70%	-2.72%
EQA-SEGA-05-04	33.97	36.57	33.23	−7.11%	+2.23%
EQA-SEGA-05-06	26.56	28.56	26.68	−7.00%	-0.45%
EQA-SEGA-05-09	30.26	33.39	30.58	−9.37%	−1.05%
Mean	29.57	32.16	29.69	−8.05%	−0.40%

A negative bias against reference laboratory accounts for better performance of our multiplex assay

## Discussion

The aim of our study was to implement a syndromic approach based upon the use of a multiplex real time RT-PCR assay to facilitate rapid diagnosis of dengue-like syndromes in Reunion Island. This syndromic approach was in phase with regional health agency preoccupations because of the recurrent occurrences of leptospirosis but also taking into account the unprecedented major CHIKV outbreak in 2005–2006 and the regular number of clustered cases of DENV [22]. On the one hand, the use of a syndromic panel is appealing for economic reasons. It is cost saving due to the use of lower amount of enzyme than in simplex reactions. The multiplex assay requires less handle-time and it is subjected to lower risk of undesirable cross-reaction contaminations. On the other hand, multiplex assays have been criticized for a loss of sensitivity compared to their simplex counterparts. Stacking up several validated simplex PCR systems into one multiplex reaction can lead to pitfalls unless precautions are taken by reducing size of the different probes and by checking possible oligonucleotide mismatches. Given the co-circulation of CHIKV and DENV in several parts of the world, duplex real time RT-PCR protocols have been set up using unspecific SYBR green fluorescence detection method [23, 24] or fluorescence labelled specific probes [25]. To our knowledge, this is the first time that a multiplex approach is designed to simultaneously detect for the presence of CHIKV, DENV and pathogenic leptospires in the same human sample. A multiplex assay for DENV, malaria and leptospirosis was published in 2007 but 16S rRNA genomic sequence used to target leptospiral genome was not specific to pathogenic *Leptospira* species [26]. Other multiplex assays in the field of arbovirus diagnosis are available for dengue typing [18, 23, 27–29].

Dealing with multiplex set up assays, we paid a critical attention to sensitivity. In human CHIKV infection, plasma viral load is usually high during the acute phase and this is probably due to the high rate of replication known for alphaviruses.

In DENV infections, Cts are usually higher corresponding with lower viral load and, therefore, urging for a more sensitive test. Our assay performed well and we decided to greatly reduce the final reaction volume to 10 μL instead of 20 μL and without affecting assay sensitivity.

CHIKV detection was done using unmodified Pastorino’s protocol [16]. In our multiplex assay, only one higher Ct difference was evidenced when compared to simplex assay. It should be noted that half of total RNA was used in multiplex assay when compared to simplex format.

DENV assay was modified to achieve better sensitivity. We started from Leparc-Goffard’s protocol and used modified primers with a newly designed probe [18]. By the time we implemented this DENV assay design in our routine testing, Alm et al. published in December 2014 a simplex RT-PCR method for DENV detection [30]. Their probe was very similar to ours. Possible consensus sequences in 3′NC region of all types DENV genome are very limited taking into account “no mismatch” design of the probe. Such design offered guaranties in sensitive detection using the fluorescent probe. Alm and colleagues have used an MGB™ probe that fits a 16 nucleotide long sequence. In our assay we have designed a 14 nucleotide long probe and incorporated LNA™ nucleotides to fit thermal requirements of TaqMan® probe design. LNA™ probe and MGB™ probes are effective but LNA™ probes accommodate a more versatile use of fluorochromes than MGB™ probes. This is especially relevant for a multiplex use of different fluorescence labeled hydrolysis probes.

Pathogenic leptospires were well detected with our multiplex assay and targeting the genomic sequence of the 23S ribosomal RNA. Due to the absence of available EQA for leptospirosis, accuracy was not checked.

We considered the use of ABI TaqMan® Fast Virus 1-Step Master Mix as a promising approach for the detection of both DNA and RNA targets in a RT PCR formatted assay and according to the manufacturer’s instructions. This feature was used to combine in the same assay, detection for viral RNA and bacterial total nucleic acids without a loss of sensitivity for leptospirosis diagnosis. The use of this one step RT-PCR system highlighted the possible development of multiplex assays designed either for the combined detection of viral RNA and viral DNA or the dual detection of bacterial DNA and viral RNA. Hence, there is an opportunity to develop new applications of multiplex PCR assays for diagnostic purposes.

Regarding syndromic PCR panels, commercial tests are usually based on dedicated master-mixes for either DNA targets or RNA targets. The possibility to combine DNA and RNA mixes will be important to address more efficiently multiplex PCR issues.

A limitation of syndromic PCR panels is represented by the number of targets that can be simultaneously detected. Real time thermocyclers rarely handle more than 5 to 6 flurorochome detection systems leading to the use of several master-mixes containing different primers and combinations of probes in order to increase the number of detected targets. To perform such PCR detection kits, significant amounts of nucleic acid templates are required. In the presence of low amount of biological matrices such as cerebrospinal fluid or plasma neonate’s samples the use of multiplex assays especially designed to be run in a 10 μL reaction volume and requiring therefore less template is highly desirable.

In Reunion Island the usefulness of such syndrome screening test should be emphasized given the concomitant circulation of CHIKV, DENV and pathogenic leptospires during the rainy season, and leading to similar clinical symptoms in patients. It should be noted that differential diagnosis of leptospirosis is a crucial issue regarding patient’s treatment with specific antibiotherapy. During the CHIKV epidemics in la Reunion from 2005 to 2006, leptospirosis diagnosis has been underestimated leading to misclassification and leading to leptospirosis related fatal issues [31]. Several parts of Europe are of great concern since invasive mosquitoes *Aedes albopictu*s and *Aedes aegyptii* are found in a larger extent. DENV and CHIKV imported cases and limited autochthonous circulation of these virus have been consistently reported in metropolitan France. [32]. Hence, the strength of a syndrome screening test addressed herein was to detect and to follow emergence of pathogens of public health concern in new geographical areas.

## Conclusions

Molecular diagnosis of three infectious agents in patients attending our regional hospital was performed using a new in-house one-step multiplex real time RT-PCR assay. A two-year follow-up of this implemented assay led to remarkable performance to allow for the detection in human samples of CHIKV, DENV and pathogenic leptospira. Evaluation and assay’s accuracy have been successfully checked on a regular basis using CHIKV and DENV EQA. This assay offers reliable responses to pathogen identification challenges and particularly for dengue-like syndromes.

## Abbreviations

3′ NC: 3′ non coding region
CHIKV: Chikungunya virus
DENV: Dengue virus
EQA: External quality assessment
LNA™: Locked nucleic acid DNA probe
MGB™: Minor groove binder DNA probe
SEGA: “Surveillance des Epidémies et Gestion des Alertes de l’Océan Indien” for epidemiological surveillance and response in the Indian Ocean
